# BMP-9 and LDL crosstalk regulates ALK-1 endocytosis and LDL transcytosis in endothelial cells

**DOI:** 10.1074/jbc.RA120.015680

**Published:** 2021-01-13

**Authors:** Bo Tao, Jan R. Kraehling, Siavash Ghaffari, Cristina M. Ramirez, Sungwoon Lee, Joseph W. Fowler, Warren L. Lee, Carlos Fernandez-Hernando, Anne Eichmann, William C. Sessa

**Affiliations:** 1 Department of Pharmacology, Yale University School of Medicine, New Haven, Connecticut, USA; 2 Vascular Biology and Therapeutics Program, Yale University School of Medicine, New Haven, Connecticut, USA; 3 Department of Laboratory Medicine and Pathobiology, University of Toronto, Toronto, Ontario Canada; 4 Department of Comparative Medicine, Yale University School of Medicine, New Haven, Connecticut, USA; 5 Keenan Research Centre for Biomedical Science, St. Michael's Hospital, Toronto, Ontario, Canada; 6 Department of Biochemistry and Medicine, University of Toronto, Toronto, Ontario, Canada; 7 Department of Internal Medicine, Cardiovascular Research Center, Yale University School of Medicine, New Haven, Connecticut, USA; 8 Department of Cellular and Molecular Physiology, Yale University School of Medicine, New Haven, Connecticut, USA

**Keywords:** LDL, transcytosis, ALK-1, caveolin, signaling, cardiovascular, caveolae, low-density lipoprotein, signal transduction, endothelium

## Abstract

Bone morphogenetic protein-9 (BMP-9) is a circulating cytokine that is known to play an essential role in the endothelial homeostasis and the binding of BMP-9 to the receptor activin-like kinase 1 (ALK-1) promotes endothelial cell quiescence. Previously, using an unbiased screen, we identified ALK-1 as a high-capacity receptor for low-density lipoprotein (LDL) in endothelial cells that mediates its transcytosis in a nondegradative manner. Here we examine the crosstalk between BMP-9 and LDL and how it influences their interactions with ALK-1. Treatment of endothelial cells with BMP-9 triggers the extensive endocytosis of ALK-1, and it is mediated by caveolin-1 (CAV-1) and dynamin-2 (DNM2) but not clathrin heavy chain. Knockdown of CAV-1 reduces BMP-9–mediated internalization of ALK-1, BMP-9–dependent signaling and gene expression. Similarly, treatment of endothelial cells with LDL reduces BMP-9–induced SMAD1/5 phosphorylation and gene expression and silencing of CAV-1 and DNM2 diminishes LDL-mediated ALK-1 internalization. Interestingly, BMP-9–mediated ALK-1 internalization strongly re-duces LDL transcytosis to levels seen with ALK-1 deficiency. Thus, BMP-9 levels can control cell surface levels of ALK-1, via CAV-1, to regulate both BMP-9 signaling and LDL transcytosis.

It is believed that the initiation of atherosclerosis occurs from the subendothelial retention and accumulation of cholesterol-rich, apolipoprotein B100 (apoB-100) containing particles, primarily low density lipoprotein (LDL) (1). The phenomenon of LDL permeating the vascular endothelium has been studied for decades, and classic EM studies have shown that this occurs by transcytosis of LDL, independent of the LDL receptor (LDLR) (2). Despite the potential importance of LDL transcytosis in atherosclerotic vascular disease, the mechanisms and pathways responsible for this phenomenon have not been explored until relatively recently.

Work from our group has shown that a member of the TGF-β type 1 receptor family, activin-like kinase 1 (ALK-1), can serve as a binding protein and potential receptor for the uptake and transcytosis of LDL and very low density lipoprotein, but not oxidized LDL or high density lipoprotein across the vascular endothelium (3). Interestingly, LDL binding to ALK-1 does promote transcytosis but does not evoke canonical ALK-1–dependent signaling. In contrast, the high affinity ligands, bone morphogenetic protein-9 (BMP-9) and BMP-10, bind to ALK-1 and several TGF-β type 2 receptors to induce the phosphorylation of SMAD1/5/8 and subsequent recruitment of SMAD4 to regulate gene expression (4). Although purified BMP-9 does not compete for LDL binding to purified ALK-1, implying different binding sites for BMP-9 *versus* LDL on ALK-1, if and how BMP-9 regulates ALK-1 internalization, and impact of this on LDL uptake and transcytosis has not been systematically studied. Because fluctuations in BMP-9 plasma levels occur (5, 6) and LDL and very low density lipoprotein levels can exceed the capacity for LDL receptor hepatic clearance during hyperlipidemia, it is feasible that elevated levels LDL may impact the magnitude or duration of BMP-9 function in the vasculature.

Thus, the goal of the present study was to examine the relationship between BMP-9 and LDL as ligands for ALK-1, to discern the pathways mediating their internalization and to interrogate the actions of BMP-9 on LDL transcytosis in endothelial cells.

## Results

### BMP-9 and -10 induce internalization of ALK-1

**Figure 1:**
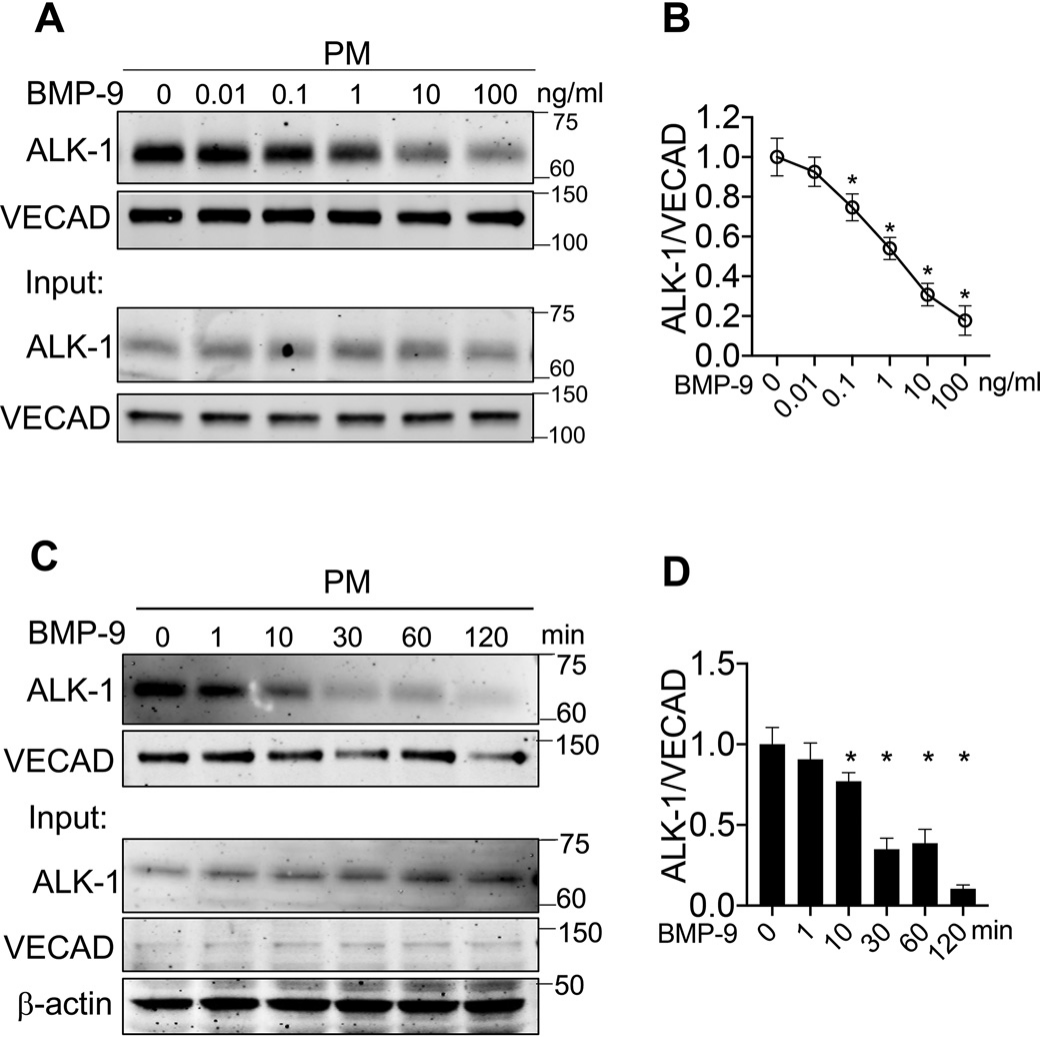
**BMP-9 triggers ALK-1 internalization.***A*, HUVEC were treated with a range of human BMP-9 for 30 min, and biotin-labeled proteins on the plasma membrane were isolated and immunoblotted for ALK-1 or VECAD. *B*, quantitative analysis of ALK-1 on the cell surface normalized to VECAD levels. *C*, HUVECs were treated with BMP-9 (10 ng/ml) for indicated times, and cell surface biotin-labeled protein analyzed. *D*, quantitative analysis of results of ALK-1 on the cell surface normalized to VECAD levels. Data represented three or four independent experiments, and quantification represents the mean ± S.E. Statistical significance was assessed by one-way ANOVA (*, *P* ≤ 0.05 when significant).

BMP-9 and -10 are the cognate ligands for the type 1 receptor, ALK-1; however, little is known about the fate of the receptor after binding BMP-9 or -10. Initially, we assessed if BMP-9 and -10 could induce internalization and endocytosis of ALK-1. HUVECs were surface labeled with a cell-impermeable biotin analog and treated with BMP-9 followed by Western blot analysis of biotin-labeled plasma membrane (PM) proteins. As seen in Fig. 1*A*, BMP-9 treatment for 30 min did not change the levels of total ALK-1 in cell lysates (*input*, *bottom panel*) but dose-dependently reduced ALK-1 levels, but not VE-cadherin (VECAD), in PM (*top panel* and quantified in Fig. 1*B*). As little as 100 pg/ml of BMP-9 triggered internalization of ∼30% of ALK-1 whereas 100 ng/ml reduced ALK-1 on the cell surface by greater than 90%. BMP-9 (10 ng/ml)–mediated internalization of ALK-1 occurred rapidly (within 1 min) and maximal internalization was achieved after 60–120 min of BMP-9 treatment (Fig. 1*C* and quantified in Fig. 1*D*). Several EC plasma membrane proteins were quantified and BMP-9 (10 ng/ml) did not affect the levels of VECAD, VEGFR2, or CD31 (Fig. S1A and quantified in Fig. S1B). Similar data were obtained using BMP-10 as a ligand for ALK-1; however, BMP-10 was less efficient (Fig. S1, C and D). Moreover, neither TGF-β1 (10 ng/ml) nor VEGF-A (10 ng/ml), ligands that activate endothelial cell signaling, affected ALK-1 internalization (Fig. S1, E and F). Thus, ALK-1 is selectively and efficiently internalized by specific ligands, BMP-9 and -10, in endothelial cells.

### Dissection of the endocytic pathways regulating ALK-1 internalization

**Figure 2:**
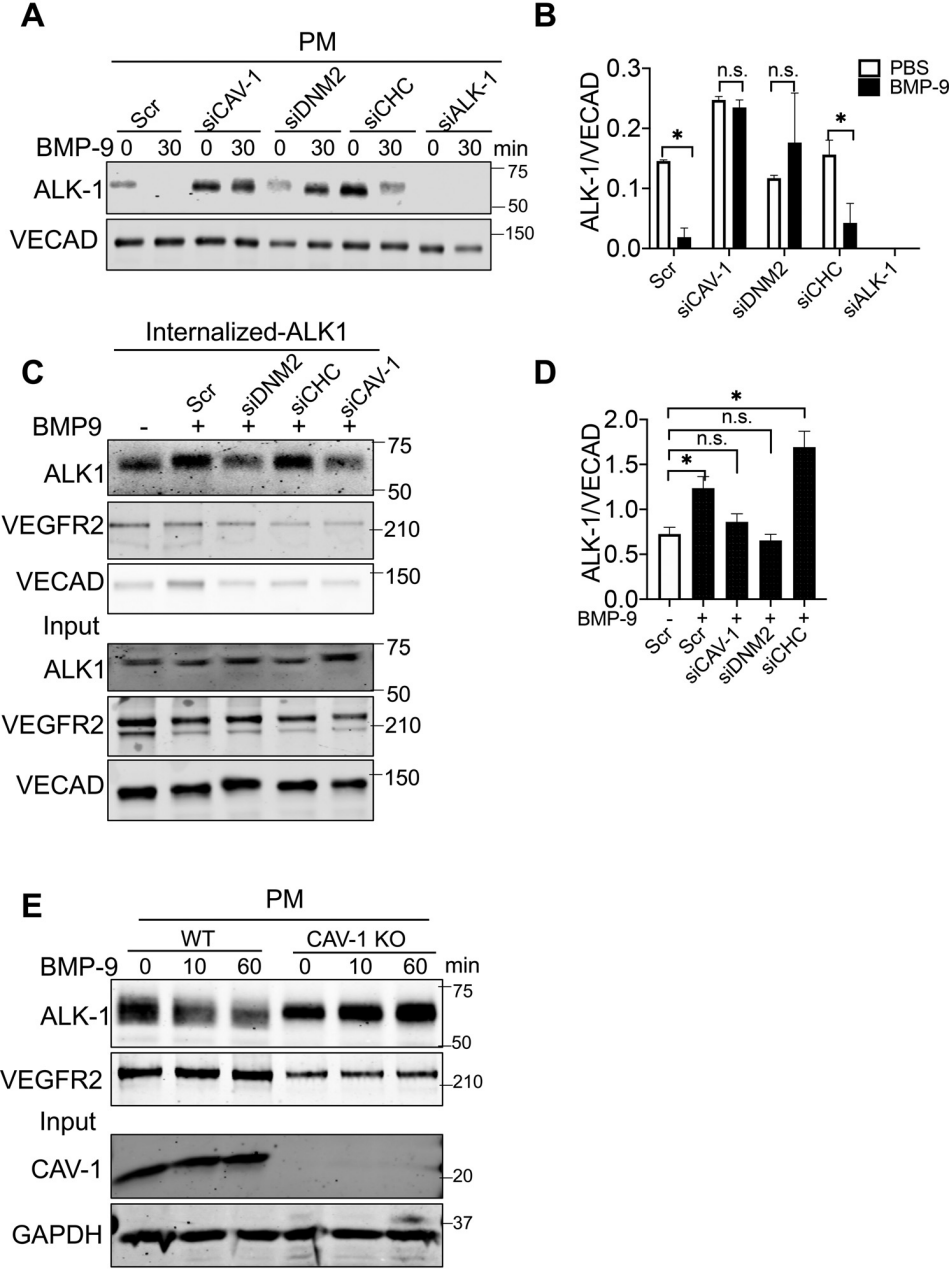
**CAV-1 and DNM2 mediate endocytosis of ALK-1 triggered by BMP-9.***A*, HUVEC were transfected with a control scrambled (*Scr*) or siRNAs against CAV-1, DNM2, CHC, and ALK-1 for 48 h and incubated in EBM2 media for 3 h before supplementation of human BMP-9 (0.5 ng/ml) for 30 min. Cells were then biotinylated and ALK-1 and VECAD detected. *B*, quantification of ALK-1 levels were normalized to surface VECAD levels. *C*, to detect internalized ALK-1, HUVEC were labeled with nonpermeable biotin on ice for 30 min, dilute acid washed, and then incubated with BMP-9 (0.5 ng/ml) for 1 h. *Top* blot reflects isolated, biotinylated proteins, and *lower* blot reflects nonenriched, total protein. *D*, quantitative analysis of internalized ALK-1 relative to VECAD. *E*, mLEC isolated from littermates of WT and CAV-1 KO mice were immortalized and mLEC were treated with murine BMP-9 (0.5 ng/ml) for the indicated times and cell surface biotinylation performed as above. Data represented three or four independent experiments. Statistical significance was assessed by one-way ANOVA (*, *P* ≤ 0.05 when significant).

Next, we sought to determine which endocytic pathway is responsible for BMP-9–mediated ALK-1 internalization. Ligand-induced endocytosis in endothelial cells can be mediated through several pathways including clathrin (clathrin heavy chain; CHC) or caveolin (CAV-1) dependent entry, both of which utilze dynamin-2 (DNM2) as the intracellular GTPase critical for membrane fission. Thus, HUVECs were treated with siRNAs to reduce levels of CAV-1, DNM2, and CHC (Fig. S2, A and B for siRNA validation) and BMP-9–mediated ALK-1 internalization from the PM assessed. As seen in Fig. 2*A* (and quantified in Fig. 2*B*), BMP-9 (0.5 ng/ml for 30 min) induced the internalization of ALK-1, an effect attenuated by knockdown of CAV-1 and DNM2, but not CHC. Interestingly, silencing of CAV-1 enhanced the basal levels of ALK-1 in PM. To examine this using a different method, the amount of internalized ALK-1 (*versus* ALK-1 remaining on the cell surface) was quantified after cleavage of biotin on the cell surface. Indeed, knockdown of DNM2 and CAV-1, but not CHC, reduced BMP-9 stimulated ALK-1 internalization (Fig. 2*C* and quantified in Fig. 2*D*). Next, we took a genetic approach to examine if the loss of CAV-1 affected ALK-1 endocytosis. Mouse lung endothelial cells (mLEC) were isolated from WT and CAV-1 knockout (CAV-1KO) mice and BMP-9–mediated ALK-1 internalization assessed. As seen in Fig. 2*E*, the loss of CAV-1 reduced BMP-9–mediated ALK-1 endocytosis. Thus, BMP-9 mediates ALK-1 internalization, in part, via a CAV-1–dependent pathway.

### CAV-1 is critical for BMP-9 signaling

**Figure 3:**
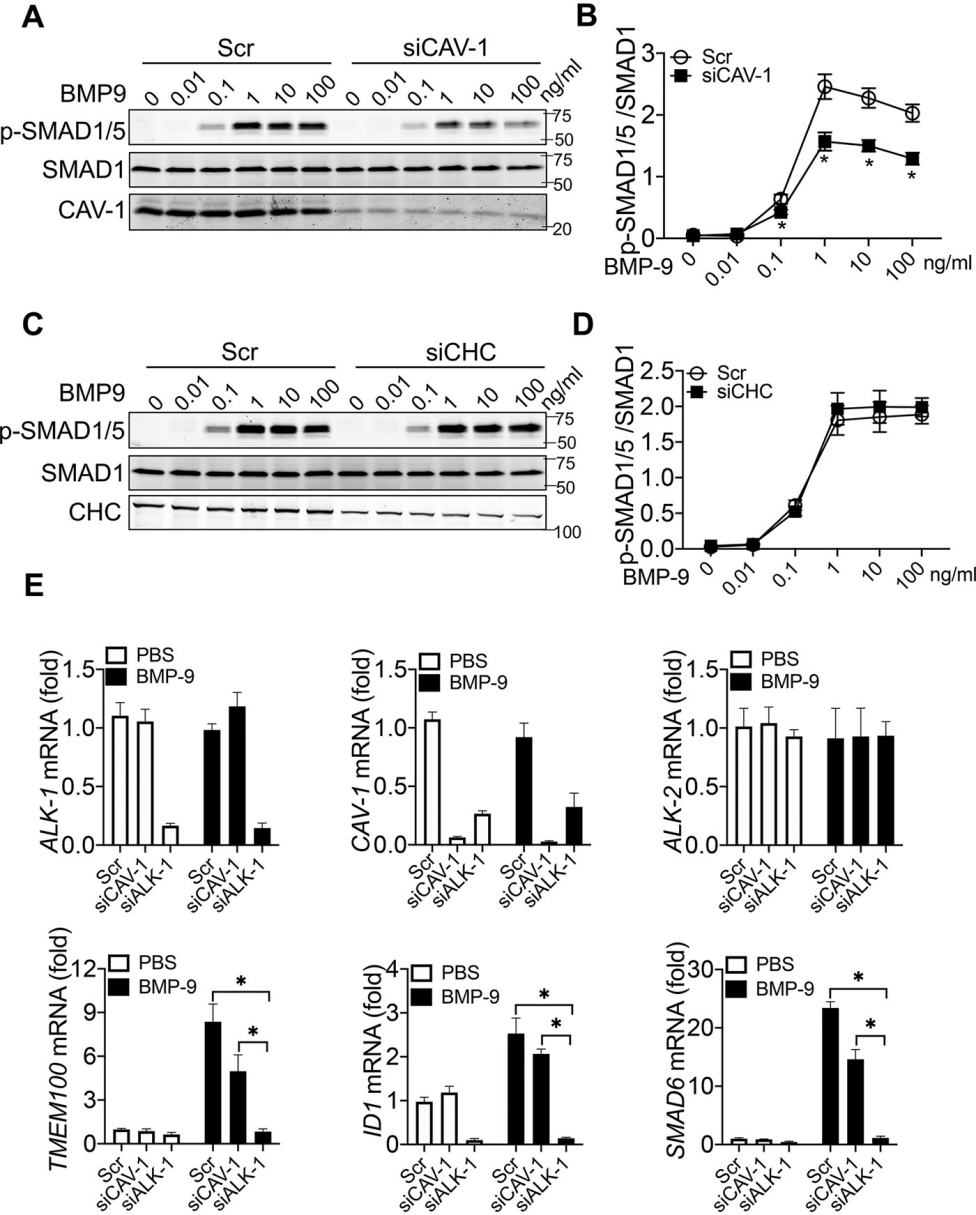
**Depletion of CAV-1, but not CHC reduces BMP-9 signaling.***A*–*D*, HUVEC were transfected with a control scrambled (*Scr*) or siRNAs against CAV-1 (*A* for blot and quantified in *B*) or CHC (*C* for blot and quantified in *D*) and incubated with BMP-9 for 0.5 h followed by immunoblotting for p-SMAD1/5 and total SMAD1. *E*, HUVEC were transfected with siRNAs and BMP-9 treated (0.5 ng/ml for 16 h) and SMAD1/5 dependent gene expression *(TMEM-100, ID1, and SMAD6)* quantified by qRT-PCR. The knockdown efficiency and specificity were measured using primers pairs for ALK-1, ALK-2, or CAV-1. Data are representative three or four independent experiments. Statistical significance was assessed by one-way ANOVA (*, *P* ≤ 0.05).

To investigate the relationship between BMP-9–induced ALK-1 internalization and signaling, BMP-9 stimulated SMAD1/5 phosphorylation and SMAD-dependent gene expression was measured. Depletion of CAV-1 (Fig. 3*A* and quantified in Fig. 3*B*) reduced BMP-9–stimulated SMAD1/5 phosphorylation, whereas depletion of CHC did not impact signaling (Fig. 3, *C* and *D*). Next, a panel of BMP-9–inducible genes were quantified in HUVEC after either CAV-1 or ALK-1 silencing. As seen in Fig. 3*E*, BMP-9 (0.5 ng/ml) induced expression of canonical SMAD1/5-dependent genes, *TMEM100, ID1*, and *SMAD6*, and this induction was reduced by CAV-1 depletion and abrogated by ALK-1 silencing. BMP-9 did not affect the expression of ALK-2 or CAV-1. Similar results were obtained with different concentrations of BMP-9 (Fig. S3, A–C). Thus, CAV-1 is critical for BMP-9–stimulated ALK-1 endocytosis, signaling, and gene expression.

### ALK-1 is colocalized and interacts with CAV-1

**Figure 4:**
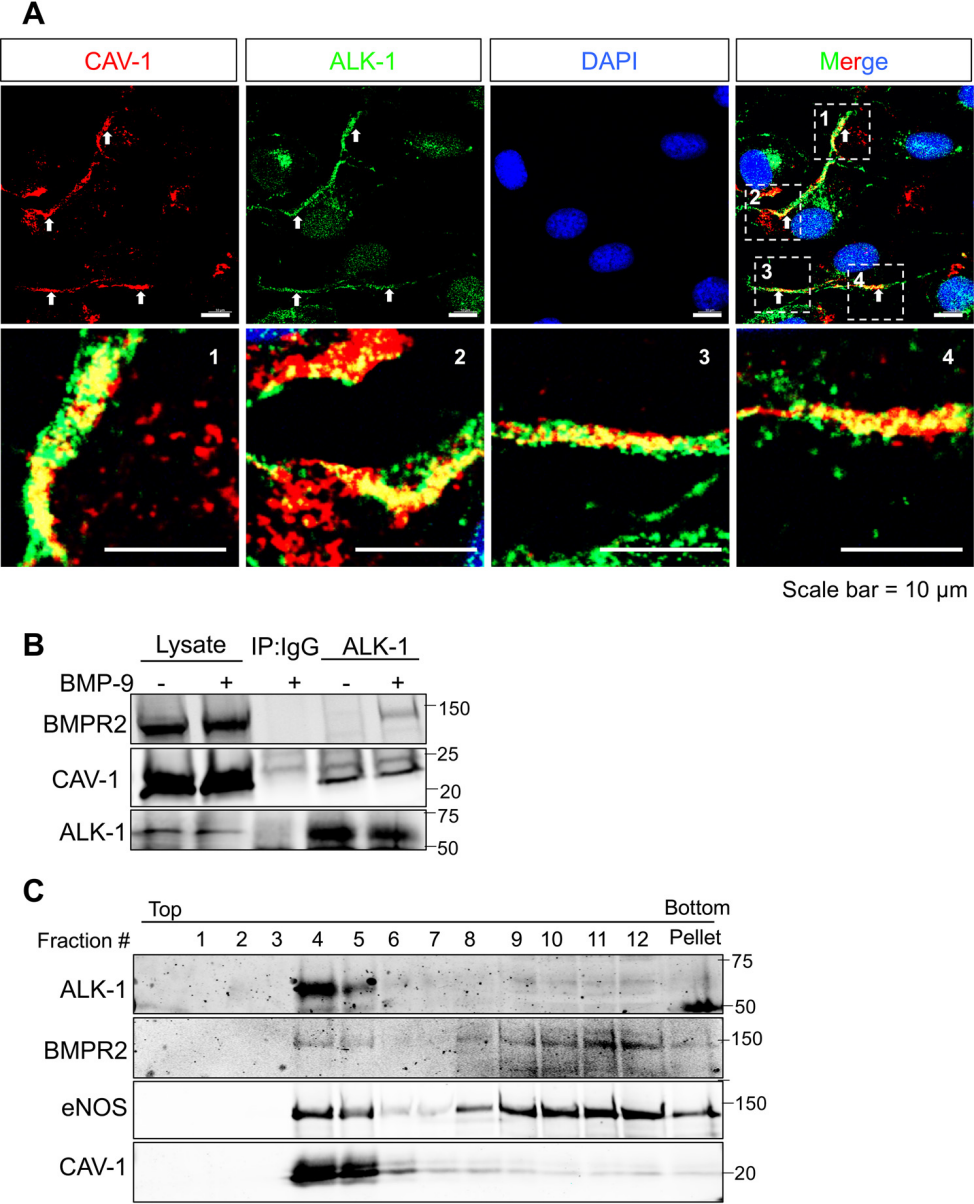
**ALK-1 colocalizes, interacts, and co-sediments with CAV-1.***A*, representative confocal images of CAV-1 (*red channel*) and ALK-1 (*green channel*) in HUVEC; DAPI was used to visualize the nucleus. Highlighted areas reflect magnified areas from four different cells in the *upper panel*. *Scale bars* = 10 μm. *B*, HUVEC were treated with BMP-9 (0.5 ng/ml) for 30 min, ALK-1 immunoprecipitated using antibody against ALK-1 or isotope-matched control IgG. *C*, membrane separation on a sucrose gradient prepared from HUVEC depicting light membranes floating in fractions 3–5 and heavy membranes in fractions 8–12.

To explore the spatial connection between CAV-1 and ALK-1, confocal microscopy was employed and endogenous CAV-1 and ALK-1 colocalize in HUVECs (Fig. 4*A*). To examine if BMP-9 impacts recruitment of BMPR2, a type 2 receptor critical for ALK-1–dependent signaling to SMAD1/5 (7), HUVECs were treated with BMP-9 for 10 min and immunocomplex formation examined by immunoprecipitation of ALK-1. As seen in Fig. 4*B*, precipitation of ALK-1 resulted in the co-association of CAV-1 (in the absence and presence of BMP-9 treatment), and this complex was not found in control precipitates with an isotype control Ab. Interestingly, precipitation of ALK-1 under BMP-9 stimulated conditions resulted in the co-association of CAV-1 and BMPR2 with the complex. Detergent-free isolation of CAV-1/lipid raft–enriched domains from HUVECs showed that ALK-1 is exclusively located in buoyant, CAV-1–enriched membranes (Fig. 4*C*; *fractions 4 and 5*), whereas BMPR2 is in both light and heavy membranes (*fractions 9–12*) similar to that seen with endothelial nitric oxide synthase (eNOS).

### LDL impairs ALK-1–dependent SMAD1/5 signaling

**Figure 5:**
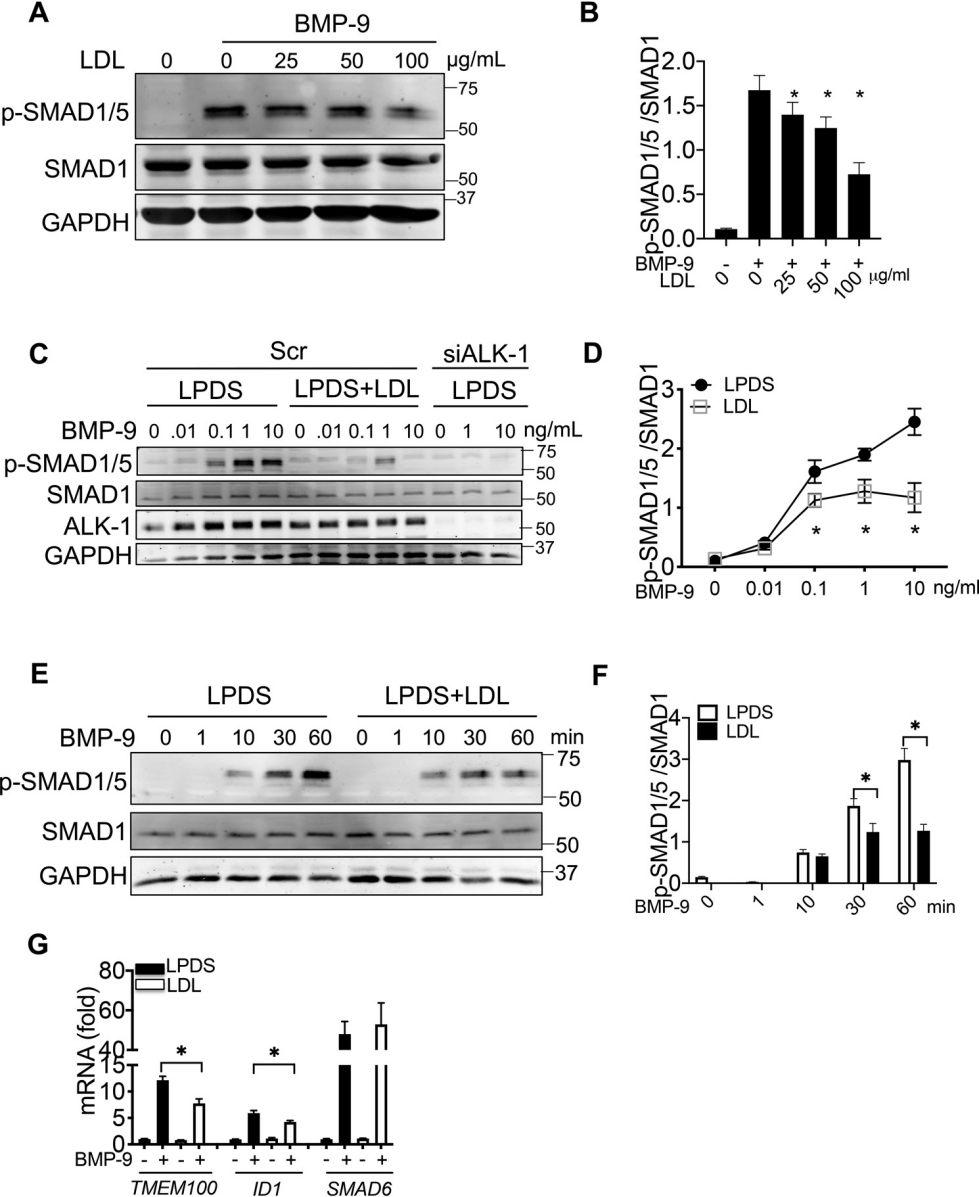
**LDL suppresses BMP-9/ALK-1 signaling.***A*, HUVECs were incubated with different amounts of LDL for 16 h, then treated with BMP-9 (0.5 ng/ml) for 30 min. *B*, quantification p-SMAD/SMAD1 from three independent experiments. *C* and *D*, HUVECs were transfected with Scr or ALK-1 siRNAs then cultured in LPDS or LPDS containing LDL (25 µg/ml) for an additional 16 h. Treated cells were then incubated with differing amounts of BMP-9 (0.01-10 ng/ml) to assess p-SMAD1/5 phosphorylation and quantified in *D*. *E* and *F*, kinetics of SMAD1/5 phosphorylation in the absence and presence of LDL. HUVECs were incubated in LPDS or in LPDS plus LDL (25 μg/ml) for 16 h and then stimulated with BMP-9 (0.5 ng/ml) for indicated time and quantified in *F*. *G*, SMAD1/5-dependent gene expression is reduced in LDL pretreated cells. HUVECs were treated with LDL (25 μg/ml for 16 h) and then incubated with BMP-9 (0.5 ng/ml) for 8 h and qPCR analysis performed. All quantitative data were from three independent experiments.

Previous work uncovered ALK-1 as receptor for native LDL transcytosis in endothelial cells. The binding occurs through apoB-100 on the LDL particle, is independent of BMP-9 binding, and does not directly activate SMAD1/5 signaling (3). Thus, we examined if LDL-mediated internalization of ALK-1 affected BMP-9 signaling to SMAD1/5. Preincubation of HUVEC in lipoprotein-depleted serum (LPDS) containing increasing concentrations of LDL resulted in dose-dependent suppression of BMP-9–mediated SMAD1/5 activation (Fig. 5*A* and quantified in Fig. 5*B*). Incubation with LDL blunted the increase in SMAD1/5 phosphorylation in response to different concentrations of BMP-9 (Fig. 5*C* and quantified in Fig. 5*D*), and this occurred in a time-dependent manner (Fig. 5*E* and quantified in Fig. 5*F*). Accordingly, the reduction in SMAD1/5 phosphorylation triggered by LDL-blunted BMP-9–induced gene expression of *TMEM100* and *ID1,* but not *SMAD6* (Fig. 5*G*).

### LDL triggers ALK-1 internalization via CAV-1 and DNM2

**Figure 6:**
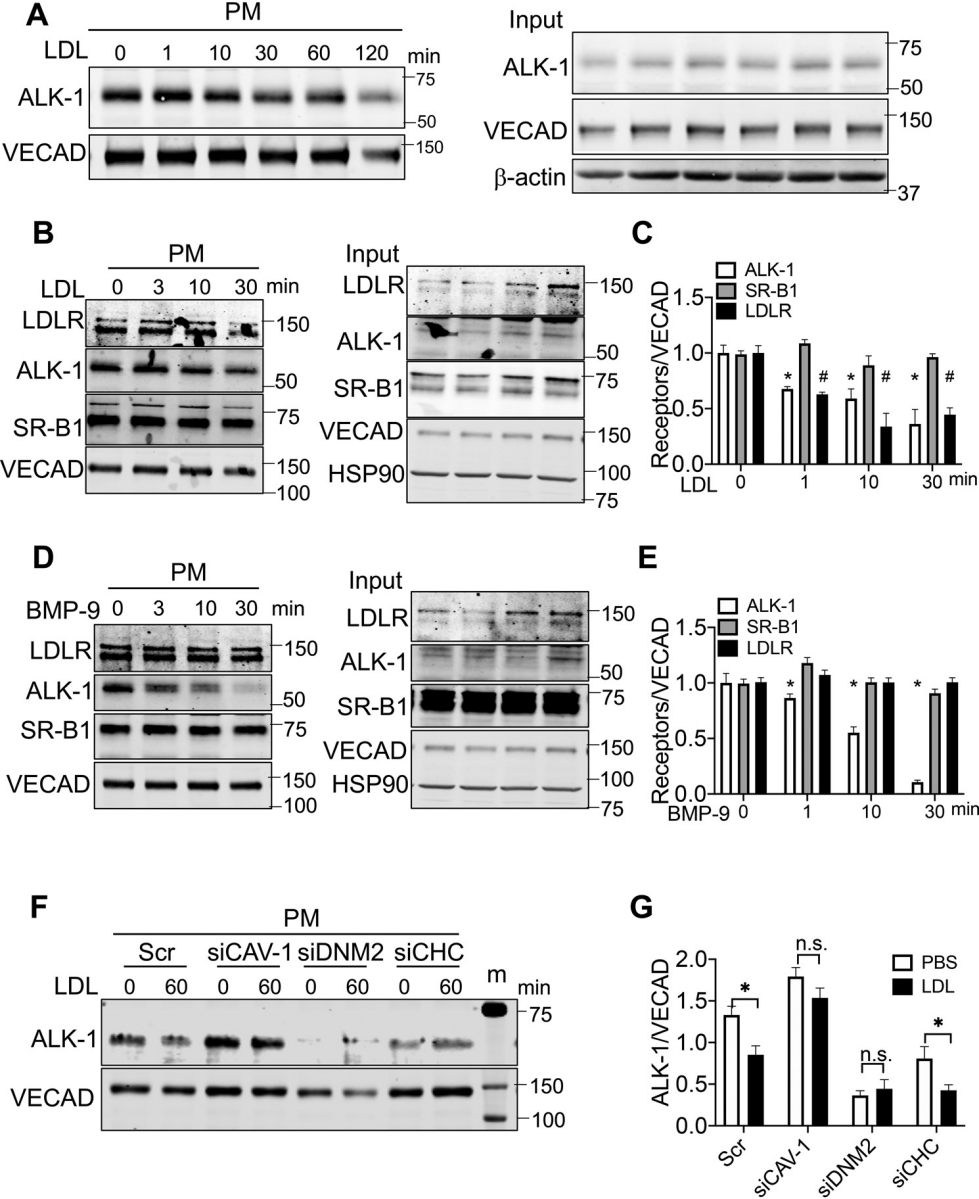
**LDL triggers the endocytosis of ALK-1.***A*, HUVECs were incubated with LDL (25 μg/ml) for the indicated times and biotinylated cell surface proteins isolated for immunoblotting. *B*–*E*, levels of cell surface LDLR, ALK-1, and SR-B1 after treatments of HUVEC with LDL (*B*, 25 μg/ml) or BMP-9 (*D*, 0.5 ng/ml) for the indicated time (0–30 min) and quantified in *C* and *E*. *F* and *G*, HUVEC were transfected with siRNAs for 48 and incubated with LDL (25 μg/ml) for 60 min; ALK-1 on the cell surface detected using biotin-labeling and the quantified *G*. Quantitative data were from three independent experiments.

Next, we tested if LDL could serve as a nonclassical ligand for ALK-1. Treatment of HUVEC with LDL (2.5–100 μg/ml for 60 min) did not result in SMAD1/5 phosphorylation whereas 0.5 ng/ml of BMP-9 robustly induced SMAD1/5 phosphorylation (Fig. S4A). These data are consistent with previous work showing that loss of function or gain of function mutants of the kinase domain of ALK-1 equally internalized LDL (3). Interestingly, LDL time-dependently reduced ALK-1 levels on the plasma membrane in HUVECs replete with serum (Fig. 6*A*) or in cells in LPDS (Fig. S4B and quantified in Fig. S4C). LDLR is a high affinity receptor for LDL on the endothelium that mediates the uptake and distribution of LDL-derived cholesterol to cells, whereas ALK-1 and SR-B1 serve as receptors for LDL transcytosis (3, 8, 9), but not for the delivery of LDL cholesterol to intracellular membranes (10). Thus, we monitored the LDL-dependent internalization of all three receptors. LDL promoted the time-dependent internalization of both LDLR and ALK-1, but not SR-B1 (Fig. 6*B* and quantified in Fig. 6*C*). BMP-9 treatment reduced ALK-1 protein levels in the PM without influencing LDLR or SR-B1 levels (Fig. 6*D* and quantified in Fig. 6*E*). The lack of effect of BMP-9 on LDLR levels was also confirmed by flow cytometry in nonpermeabilized cells (Fig. S4, D and E). To examine the pathway of LDL-triggered internalization of ALK-1, cells were treated with siRNAs targeting CAV-1, DNM2, and CHC. As seen in Fig. 6*F* and quantified in Fig. 6*G*), reductions of CAV-1 and DNM2, but not CHC, reduced LDL-mediated ALK-1 endocytosis.

### Reducing ALK-1 on the plasma membrane with BMP-9 mitigates LDL transcytosis and BMP signaling in vivo

**Figure 7:**
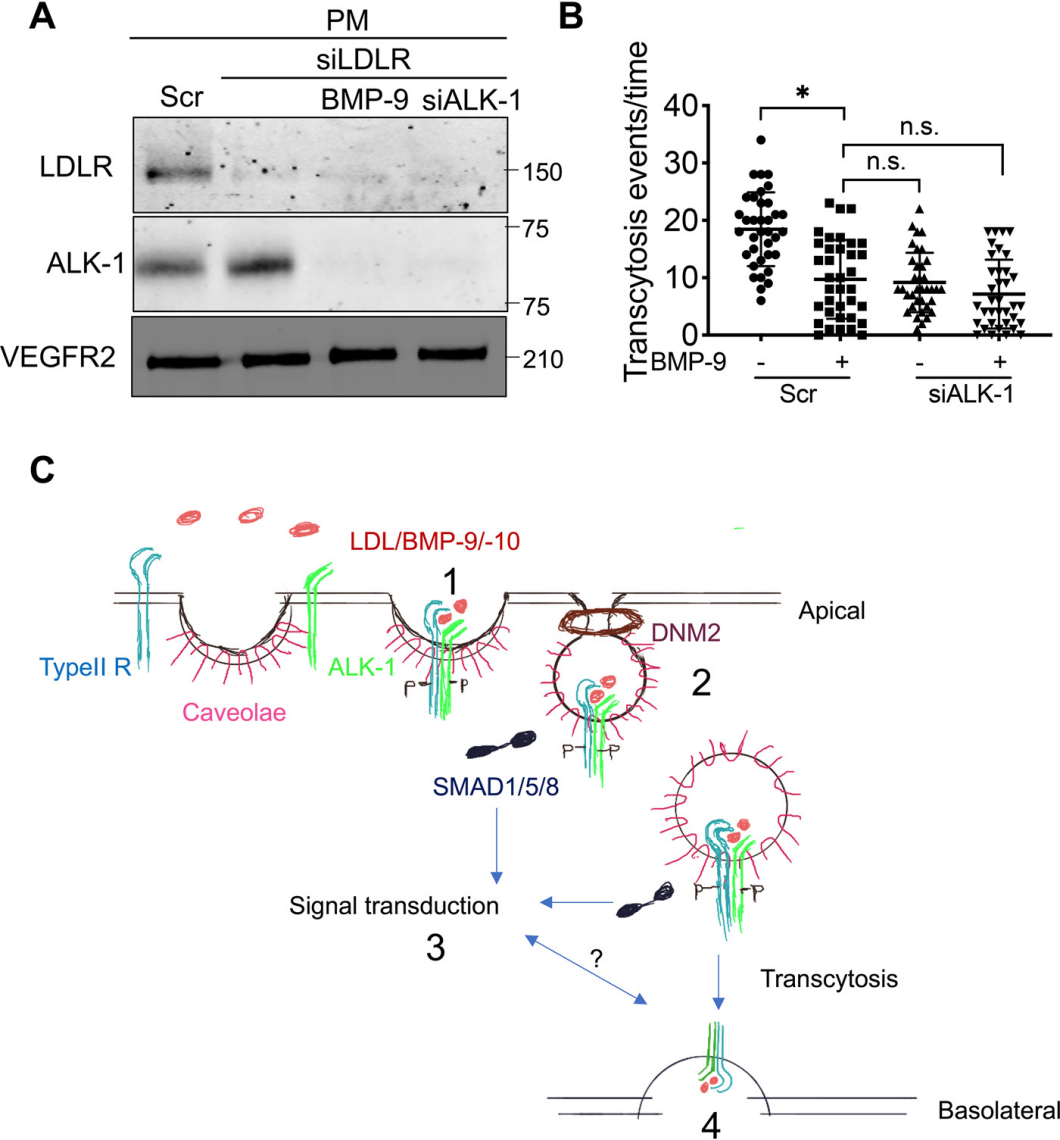
**BMP-9 treatment blunts LDL transcytosis in an ALK-1 dependent manner.***A*, HCAECs were treated with Scr or LDLR siRNAs combined BMP-9 (10 ng/ml for 1 h) treatment or ALK-1 knockdown. *K_D_* was significantly reduced. ALK-1 protein in the plasma membrane assayed by biotin-pulldown. *B*, TIRF microscopy of basolateral transcytosis events in HCAEC under the above conditions. Combined data represent three independent experiments with 12 cells per/group/time. Statistical significance was assessed by one-way ANOVA (*, *P* ≤ 0.05). *C*, model depicting crosstalk between BMP-9, LDL, and ALK-1. BMP-9/10 and LDL can bind ALK-1 that is localized in caveolae (1) inducing its internalization. DNM2 (2) is critical for ALK-1 endocytosis. Once internalized, BMP-9/ALK-1 can signal to SMADs inducing endothelial cell quiescence (3) or the LDL-ALK-1 complex can be transcytosed across the endothelium (4). How ALK-1 decides to signal *versus* transcytose LDL is not known.

Previous work has shown that ALK-1, in part, mediates LDL transcytosis across the endothelium. During hypercholesterolemia, the LDLR receptor is tonically down-regulated and transcytosis occurs independent of the LDLR via ALK-1 or SR-B1 (10). Thus, we used LDLR-depleted EC to assess the effects of BMP-9 *versus* ALK-1 silencing on LDL transcytosis (Fig. 7*A*). Human coronary arterial ECs (HCAEC) were treated with BMP-9 (10 ng/ml for 60 min) to reduce ALK-1 on the plasma membrane followed by quantifying DiI-LDL transcytosis by total internal reflectance microscopy (TIRF). BMP-9 treatment markedly reduces LDL transcytosis, and this effect is not further reduced in cells depleted of ALK-1 (Fig. 7*B*). Thus, BMP-9–induced endocytosis of ALK-1 blunts LDL-mediated transcytosis in EC. Both of BMP-9 and LDL could mediate ALK-1 internalization, which is the key step to initiate SMAD1 signaling or transcytosis (Fig. 7*C*).

## Discussion

The central goal of this study was to explore the potential crosstalk between BMP-9 and LDL as proteins that bind to ALK-1. Herein, we describe a BMP-9–mediated internalization pathway for ALK-1 that is necessary for SMAD1/5 phosphorylation and subsequent gene expression. BMP-9–mediated ALK-1 endocytosis requires both CAV-1 and DYN2, and deficiency of CAV-1 reduces BMP-9–stimulated ALK-1 internalization, SMAD1/5 phosphorylation, and gene expression. Interestingly, LDL treatment of EC promotes ALK-1 internalization and reduces BMP-9–stimulated SMAD1/5 phosphorylation. Conversely, treatment of EC with BMP-9 markedly reduces LDL transcytosis in an ALK-1–dependent manner, implying that the levels of BMP-9 may regulate LDL transcytosis and ultimately the extent of atherosclerosis.

The roles of BMP-9 and -10 in the cardiovascular system have largely focused on their important roles in arteriovenous differentiation during development and the pulmonary vasculature largely due to human genetic evidence showing that loss of function mutations in ALK-1, or its coreceptors, lead to hereditary hemorrhagic telangiectasia and/or pulmonary hy-pertension. In mice and humans, the loss of ALK-1 reduces endothelial cell quiescence, leading to excessive activation of signaling pathways promoting vascular disease (4, 11). In the context of pulmonary hypertension, BMP-9 agonism or antagonism has been shown to exert beneficial effects in models of pulmonary hypertension (12, 13). In humans with atherosclerosis, *ALK-1* mRNA levels are increased in lesions (14) and inhibition of BMP signaling *in vivo* reduces the extent of atherosclerotic plaques (15, 16). Previous work by our group has shown acute, conditional EC-specific deletion of ALK-1 reduces LDL uptake in mice lacking the LDLR (3). Because BMP-9 and -10 are high affinity ligands for ALK-1 and their levels diminish with age (5), it is feasible that the elevated levels of LDL during hypercholesterolemia may bind to ALK-1, resulting in its transcytosis and subendothelial retention of LDL.

Interestingly, in the present study, we show that CAV-1 is necessary for both LDL and BMP-9–mediated ALK-1 endocytosis. This is compelling since it is well established that the loss of CAV-1 in the endothelium reduces LDL uptake, transcytosis, and strongly attenuates atherosclerosis (17, 18, 19). The loss of CAV-1 reduces LDL uptake in isolated aortas and intact arteries *in vivo* and quantitatively reduces apoB100 levels in the vessel wall as measured using several methodologies tracking endogenous and exogenous LDL particles (17, 19). Previous work has shown that ALK-1 can be localized in caveolae (20). Here, we extend these finding and show that ALK-1 can be immunoisolated in complex with CAV-1 and the type 2 receptor, BMPR2. More importantly, the loss of CAV-1 reduces, but does not abolish, BMP-9 signaling, suggesting that caveolae and/or CAV-1 negatively regulates ALK-1 signaling at the level of the plasma membrane. The only other work demonstrating a role for the endocytic machinery in BMP signaling in blood vessels is a report showing that the clathrin adaptor, disabled homolog 2 (DAB2), is necessary for the pro-angiogenic function of BMP-2 during zebrafish vascular development (21). Suppression of ALK-1 endocytosis by depleting CAV-1 or DNM-2 in our study differs from other negative regulatory pathways that rely on termination of BMP-9 signaling by inhibitory SMADs and protein phosphatases (4). In the present study, we show that treatment with BMP-9 to promote extensive ALK-1 internalization (>80%) markedly reduces LDL transcytosis to levels comparable to the loss of ALK-1, implying that elevated BMP-9 levels may be atheroprotective and that the loss of ALK-1 in caveolae would reduce LDL transcytosis. Recent papers have identified the lipoprotein scavenger receptor SR-B1 as an additional candidate regulating LDL transcytosis (8, 9); however, treatment with BMP-9 had no effect on SR-B1 internalization, suggesting there are likely overlapping, redundant pathways that mediate LDL transcytosis.

Thus, our data are consistent with BMP-9 and LDL using the same pathway to trigger ALK-1 internalization. However, once internalized, the ligand functions are distinct. BMP-9–mediated internalization is critical for signaling to SMADs whereas LDL-mediated internalization mediates transcytosis, and molecular mechanisms of receptor sorting post internalization are not known. BMP-9 is mainly produced in liver by hepatocytes and intrahepatic biliary epithelial cells, and 60% of it is in the active form in the human circulation. BMP-9 also inhibits angiogenic growth factor (VEGF and FGF) signaling and has been described as a quiescence factor in developing mice (6, 22). In addition, atheroprotective laminar shear stress in large vessels sensitizes the endothelium to the actions of BMP-9 (23) and may be sufficient to trigger ALK-1 internalization to further lower the risk of LDL transcytosis. Future experiments designed to preserve the quiescent actions of BMP-9 and -10 while neutralizing LDL binding to ALK-1 will permit dissection of this interesting pathway *in vivo*.

## Experimental procedures

### Cell culture

HUVECs were obtained from the Yale School of Medicine, Vascular Biology and Therapeutics Core facility. HCAEC were purchased from Lonza (CC-2585). mLEC isolation was described (3): briefly, 3-week-old mice were euthanized using ketamine/xylazine; collect and pool lungs for 3 mice per group; after collagenase I digestion, CD31+ magnetic beads were applied to isolate endothelial cells. After recovery and growth, mLEC from littermates' WT or CAV-1KO were used for experiments. All of these endothelial cells were cultured in EGM-2 media (Lonza) with 10% fetal bovine serum (FBS), penicillin/streptomycin and glutamine (2.8 mm) in a 37°C incubator with 5% CO_2_ supply. Human LDL and DiI-LDL were obtained from Kalen Biomedical; BMP-9 (human and mouse) was bought from R&D Systems.

### Biotin labeling of plasma membrane

Biotin labeling was applied here to detect and quantify the mobility of receptors. In brief, for surface labeling: after the 30-min incubation on ice with a 1 mm EZ-link-sulfo-NHS-S-S-biotin solution (Pierce 21331), cells were washed with a 50 mm glycine/PBS solution, pH 7.2, and subsequently lysis in immunoprecipitation buffer. For detecting the endocytosis of the receptors, the biotin-labeled cells were treated with BMP-9 or LDL for indicated time in 37°C incubator with 5% CO_2_ supply; the biotin-labeled receptors remaining on the cell surface were stripped using GSH stripping solution (50 mm GSH, 75 mm NaCl, 1 mm EDTA, 75 mm NaOH, and 10% FBS) 15 min on ice. Following centrifugation, protein concentration was measured, Neutravidin Protein agarose beads (Pierce 29200) were added and incubation was at 4°C overnight. Flow-through fractions were collected after the incubation period following a 2000 rpm spin. The beads were then washed several times in lysis buffer prior to immunoblotting.

### Lipid raft isolation

Detergent-free lipid raft isolation was performed as described before (24). In brief, HUVECs were grown to confluence in 15 cm plates. After washing three times with ice-cold PBS, cells were scraped with 1 ml ice-cold sodium carbonate (500 mmol/liter, pH 11) supplied with a mixture of protease inhibitors, then homogenized and sonicated. Protein was loaded into the bottom of polycarbonate tubes and adjusted to 45% sucrose (w/v) by the addition of 2 ml of 90% sucrose prepared in 25 mmol/liter MES (2-(*N*-morpholino) ethanesulfonic acid; pH 6.5, 0.5 M NaCl). A 5 to 35% discontinuous sucrose gradient was formed (4 ml of 35% sucrose and 4 ml of 5% sucrose, both in MES containing 250 mmol/liter sodium carbonate) and centrifuged at 35,000 rpm for 18 h in a SW40 rotor (Beckman Coulter). Twelve gradient fractions (1 ml) and a pellet were collected from the top and mixed with SDS-PAGE loading buffer and equal volumes of each fraction were loaded, separated by SDS-PAGE, and protein distribution assessed by immunoblotting.

### Immunoblotting

Cells or tissues were lysed on ice with ice-cold lysis buffer containing 50 mm Tris-HCl, pH 7.4, 0.1 mm EDTA, 0.1 mm EGTA, 1% Nonidet P-40, 0.1% sodium deoxycholate, 0.1% SDS, 100 mm NaCl, 10 mm NaF, 1 mm sodium pyrophosphate, 1 mm sodium orthovanadate, 1 mm Pefabloc SC, and 2 mg/ml protease inhibitor mixture (Roche Diagnostics) and samples prepared. Total protein (25 µg) was loaded into SDS-PAGE followed by transfer to nitrocellulose membranes. Immunoblotting was performed with the following primary antibodies: LDLR (ab30532, Abcam), SR-B1(ab137829, Abcam), ALK-1 (70R-49334, Fitzgerald), DNM2 (ab65556, Abcam), GAPDH (2118, Cell Signaling Technology), eNOS (sc-7271, Santa Cruz Biotechnology), BMPR2 (612292, BD Biosciences), CAV-1 (610060, BD Biosciences), β-actin (sc-69879, Santa Cruz Biotechnology), HSP90 (610419, BD Biosciences). LI-COR compatible fluorescent-labeled secondary antibodies (LI-COR Biosciences). Bands were visualized on the Odyssey CLx platform (LI-COR Biosciences). Quantifications were based on densitometry using ImageJ.

### Immunofluorescence

As described in our previous study (3), confluent HUVECs (P4) were washed three times with PBS, following fixation for 10 min in PFA (4%); thereafter blocked in 5% BSA for 1 h at room temperature. The cells were incubated with antibodies against ALK-1 (AF370, R&D Systems) and CAV-1 (610406, BD Biosciences) with a dilution of 1:50 in blocking buffer at 4°C for overnight. Secondary antibodies were introduced using a standard immunofluorescence protocol and nuclei stained with DAPI (1:4000 dilution). Images were taken on a confocal microscope (SP5, Leica).

### Flow cytometry

The surface LDLR was measured using FACS (3). Briefly, HUVECs were gently detached from culture dish using 0.1% EDTA in PBS. Cells were then collected and labeled with anti-LDLR antibody or mouse IgG2b as control; 1 h post incubation, the cells were washed following with secondary antibody incubated for additional 30 min. Cells were measured by LSRII (BD Biosciences) flow cytometer and analyzed using FlowJo.

### Quantitative real-time PCR analysis

In brief, RNAs from cells or tissues were isolated using the RNeasy Plus Kit (Qiagen). Thereafter, 0.5 μg RNA/sample was retrotranscribed with the iScript cDNA Synthesis Kit (Bio-Rad). Real-time quantitative PCR (qPCR) reactions were performed in duplicate using the CFX-96 Real Time PCR system (Bio-Rad). Quantitative PCR primers (Quantitect primer assays) were obtained from Qiagen. Fold changes were calculated using the comparative CT method.

### Total internal reflection fluorescence–based transcytosis of fluorescently labeled LDL

LDL transcytosis by confluent primary HCAECs was measured by TIRF microscopy. Briefly, cells were placed in a live cell imaging chamber and treated with 20 µg/ml DiI-LDL in cold HPMI media for 10 min at 4°C to allow binding without internalization. Cells were then washed twice with cold PBS+ to remove unbound LDL and warm HPMI was added. Cells were incubated on the live cell imaging stage at 37°C for 2 min before initial image acquisition. Confluent regions of the monolayer were selected after staining with NucBlue Live ReadyProbes Reagent (Thermo Fisher) and TIRF microscopy of the basal membrane was performed to visualize exocytosis. TIRF microscopy was performed on an Leica DMi8 microscope with 63×/1.47 (O) objectives, 405 nm, 488 nm, 561 nm, and 637 nm laser lines, 450/50, 525/50, 600/50, 610/75, and 700/75 emission filters and run with Quorum acquisition software (Quorum, Canada). Microscope settings were kept constant between conditions. For each coverslip, 10–15 videos of 150 frames (100 ms exposure) were captured. Quantification of LDL transcytosis was performed and has been described in detail previously (25).

### Statistics

Statistical differences were measured with an unpaired 2-sided Student's *t* test or 1-way ANOVA with Bonferroni correction for multiple comparisons. A value of *p* ≤ 0.05 was considered statistically significant. Data analysis was performed with GraphPad Prism software (GraphPad, San Diego, CA).

## Data availability

All data are available in the manuscript and supporting information.

10.13039/100000050HHS | NIH | National Heart, Lung, and Blood Institute (NHLBI) (R35HL139945) to William C. Sessa
10.13039/100000050HHS | NIH | National Heart, Lung, and Blood Institute (NHLBI) (PO1HL107205) to William C. Sessa
10.13039/100000968American Heart Association (AHA) (MERIT) to William C. Sessa
10.13039/100000050HHS | NIH | National Heart, Lung, and Blood Institute (NHLBI) (R35HL135820) to Carlos Fernandez-Hernando
10.13039/501100001804Canada Research Chairs (Chaires de recherche du Canada) (PJT-168947) to Warren L. Lee

## References

[1] Tabas I., Williams K.J., Borén J. (2007). Subendothelial lipoprotein retention as the initiating process in atherosclerosis: Update and therapeutic implications. Circulation.

[2] Vasile E., Simionescu M., Simionescu N. (1983). Visualization of the binding, endocytosis, and transcytosis of low-density lipoprotein in the arterial endothelium in situ. J. Cell Biol.

[3] Kraehling J.R., Chidlow J.H., Rajagopal C., Sugiyama M.G., Fowler J.W., Lee M.Y., Zhang X., Ramírez C.M., Park E.J., Tao B., Chen K., Kuruvilla L., Larriveé B., Folta-Stogniew E., Ola R. (2016). Genome-wide RNAi screen reveals ALK1 mediates LDL uptake and transcytosis in endothelial cells. Nat. Commun.

[4] Goumans M.J., Zwijsen A., Ten Dijke P., Bailly S. (2018). Bone morphogenetic proteins in vascular homeostasis and disease. Cold Spring Harb. Perspect. Biol.

[5] Bidart M., Ricard N., Levet S., Samson M., Mallet C., David L., Subileau M., Tillet E., Feige J.J., Bailly S. (2012). BMP9 is produced by hepatocytes and circulates mainly in an active mature form complexed to its prodomain. Cell Mol. Life Sci.

[6] David L., Mallet C., Keramidas M., Lamandé N., Gasc J.M., Dupuis-Girod S., Plauchu H., Feige J.J., Bailly S. (2008). Bone morphogenetic protein-9 is a circulating vascular quiescence factor. Circ. Res.

[7] David L., Mallet C., Mazerbourg S., Feige J.J., Bailly S. (2007). Identification of BMP9 and BMP10 as functional activators of the orphan activin receptor-like kinase 1 (ALK1) in endothelial cells. Blood.

[8] Huang L., Chambliss K.L., Gao X., Yuhanna I.S., Behling-Kelly E., Bergaya S., Ahmed M., Michaely P., Luby-Phelps K., Darehshouri A., Xu L., Fisher E.A., Ge W.P., Mineo C., Shaul P.W. (2019). SR-B1 drives endothelial cell LDL transcytosis via DOCK4 to promote atherosclerosis. Nature.

[9] Armstrong S.M., Sugiyama M.G., Fung K.Y., Gao Y., Wang C., Levy A.S., Azizi P., Roufaiel M., Zhu S.N., Neculai D., Yin C., Bolz S.S., Seidah N.G., Cybulsky M.I., Heit B. (2015). A novel assay uncovers an unexpected role for SR-BI in LDL transcytosis. Cardiovasc. Res.

[10] Zhang X., Sessa W.C., Fernandez-Hernando C. (2018). Endothelial transcytosis of lipoproteins in atherosclerosis. Front. Cardiovasc. Med.

[11] Li W., Salmon R.M., Jiang H., Morrell N.W. (2016). Regulation of the ALK1 ligands, BMP9 and BMP10. Biochem. Soc. Trans.

[12] Tu L., Desroches-Castan A., Mallet C., Guyon L., Cumont A., Phan C., Robert F., Thuillet R., Bordenave J., Sekine A., Huertas A., Ritvos O., Savale L., Feige J.J., Humbert M. (2019). Selective BMP-9 inhibition partially protects against experimental pulmonary hypertension. Circ. Res.

[13] Long L., Ormiston M.L., Yang X., Southwood M., Gräf S., Machado R.D., Mueller M., Kinzel B., Yung L.M., Wilkinson J.M., Moore S.D., Drake K.M., Aldred M.A., Yu P.B., Upton P.D. (2015). Selective enhancement of endothelial BMPR-II with BMP9 reverses pulmonary arterial hypertension. Nat. Med.

[14] Yao Y., Zebboudj A.F., Torres A., Shao E., Boström K. (2007). Activin-like kinase receptor 1 (ALK1) in atherosclerotic lesions and vascular mesenchymal cells. Cardiovasc. Res.

[15] Derwall M., Malhotra R., Lai C.S., Beppu Y., Aikawa E., Seehra J.S., Zapol W.M., Bloch K.D., Yu P.B. (2012). Inhibition of bone morphogenetic protein signaling reduces vascular calcification and atherosclerosis. Arterioscler. Thromb. Vasc. Biol.

[16] Yao Y., Bennett B.J., Wang X., Rosenfeld M.E., Giachelli C., Lusis A.J., Boström K.I. (2010). Inhibition of bone morphogenetic proteins protects against atherosclerosis and vascular calcification. Circ. Res.

[17] Fernández-Hernando C., Yu J., Suárez Y., Rahner C., Dávalos A., Lasunción M.A., Sessa W.C. (2009). Genetic evidence supporting a critical role of endothelial caveolin-1 during the progression of atherosclerosis. Cell Metab.

[18] Frank P.G., Lee H., Park D.S., Tandon N.N., Scherer P.E., Lisanti M.P. (2004). Genetic ablation of caveolin-1 confers protection against atherosclerosis. Arterioscler. Thromb. Vasc. Biol.

[19] Ramírez C.M., Zhang X., Bandyopadhyay C., Rotllan N., Sugiyama M.G., Aryal B., Liu X., He S., Kraehling J.R., Ulrich V., Lin C.S., Velazquez H., Lasunción M.A., Li G., Suárez Y. (2019). Caveolin-1 regulates atherogenesis by attenuating low-density lipoprotein transcytosis and vascular inflammation independently of endothelial nitric oxide synthase activation. Circulation.

[20] Santibanez J.F., Blanco F.J., Garrido-Martin E.M., Sanz-Rodriguez F., del Pozo M.A., Bernabeu C. (2008). Caveolin-1 interacts and cooperates with the transforming growth factor-β type I receptor ALK1 in endothelial caveolae. Cardiovasc. Res.

[21] Kim J.D., Kang H., Larrivée B., Lee M.Y., Mettlen M., Schmid S.L., Roman B.L., Qyang Y., Eichmann A., Jin S.W. (2012). Context-dependent proangiogenic function of bone morphogenetic protein signaling is mediated by disabled homolog 2. Dev. Cell.

[22] Larrivée B., Prahst C., Gordon E., del Toro R., Mathivet T., Duarte A., Simons M., Eichmann A. (2012). ALK1 signaling inhibits angiogenesis by cooperating with the Notch pathway. Dev. Cell.

[23] Baeyens N., Larrivée B., Ola R., Hayward-Piatkowskyi B., Dubrac A., Huang B., Ross T.D., Coon B.G., Min E., Tsarfati M., Tong H., Eichmann A., Schwartz M.A. (2016). Defective fluid shear stress mechanotransduction mediates hereditary hemorrhagic telangiectasia. J. Cell Biol.

[24] Sowa G., Pypaert M., Sessa W.C. (2001). Distinction between signaling mechanisms in lipid rafts vs. caveolae. Proc. Natl. Acad. Sci. U. S. A.

[25] Ghaffari S., Naderi Nabi F., Sugiyama M.G., Lee W.L. (2018). Estrogen inhibits LDL (low-density lipoprotein) transcytosis by human coronary artery endothelial cells via GPER (G-protein-coupled estrogen receptor) and SR-BI (scavenger receptor class b type 1). Arterioscler. Thromb. Vasc. Biol.

