# Supplementary material for: BMP-9 and LDL crosstalk regulates ALK-1 endocytosis and LDL transcytosis in endothelial cells
Source: J Biol Chem. 2021 Jan 13;295(52):18179–88. doi: 10.1074/jbc.RA120.015680 (PMC7939458; doi:10.1074/jbc.RA120.015680)

Supporting information:

Sup-Figure 1 | BMP-9 selectively alters ALK-1 internalization and BMP-10 triggers ALK-1 internalization (A) HUVEC (P4) were treated with human BMP-9 (10ng/ml) for indicated times, Biotin-assays were applied for measuring ALK-1 dynamics on the plasma membrane. The total input and biotin assays for 4 EC specific membrane proteins (ALK-1, VECAD, VEGF2 and CD31) and quantification in (B). (C) HUVEC (P4) were treated with 10 ng/ml human BMP-10 for indicated time, Biotin-assays were applied for measuring ALK-1 dynamics on the plasma membrane. (D) Quantitative amount of residual ALK-1 on the plasma membrane normalized to VECAD were performed. Data are represented 3 or 4 independent experiments. Representative immunoblotting of surface receptors following 10 ng/ml TGF-β1 (E) or 10 ng/ml VEGF-A (F) treatment for indicated times. Statistical significance was assessed by one-way ANOVA (*$P \leq 0.05$).

Sup-Figure 2 | siRNA knockdown efficiency. HUVEC (P4) were transfected with siRNAs against scramble or ALK-1 (A) or CAV-1, DNM2, CHC (B) for 48 hrs. Data shown are representative and experiments were repeated in an additional 3 or 4 independent experiments.

Sup-Figure 3 | Regulations of BMP-9/ ALK-1 signaling by DNM2-CAV-1 complex. (A) 48 hrs post-transfection of siRNAs of CAV-1, ALK-1 or scrambled, HUVEC were treated with BMP-9 (0, 0.5, 10 ng/ml) for an additional 16 hrs. Total RNA was extracted for qPCR analysis using the primers against *TMEM100, SMAD6* and *ID1*. (*$P \leq 0.05$)

Sup-Figure 4 | LDL impacts BMP-9 signaling. (A) HUVEC were incubated with increasing amounts of LDL (0-100 μg/ml) or BMP-9 (0.5 ng/ml) for 1 hr before collecting for immunoblotting. (B) After overnight incubation of LDL (25 μg/ml), HUVEC were then stimulated with 0.5 ng/mL BMP-9 for 10 or 60 minutes and the receptors on the cell surface were assayed by the biotin-pull down assay and data were quantified from

multiple experiments (D). HUVEC were treated with BMP-9 (0.5 ng/ml) for 10 minutes or 1 hr, and LDLR on the plasma membrane was assayed using FACS and quantified (E).

Supp Figure 1

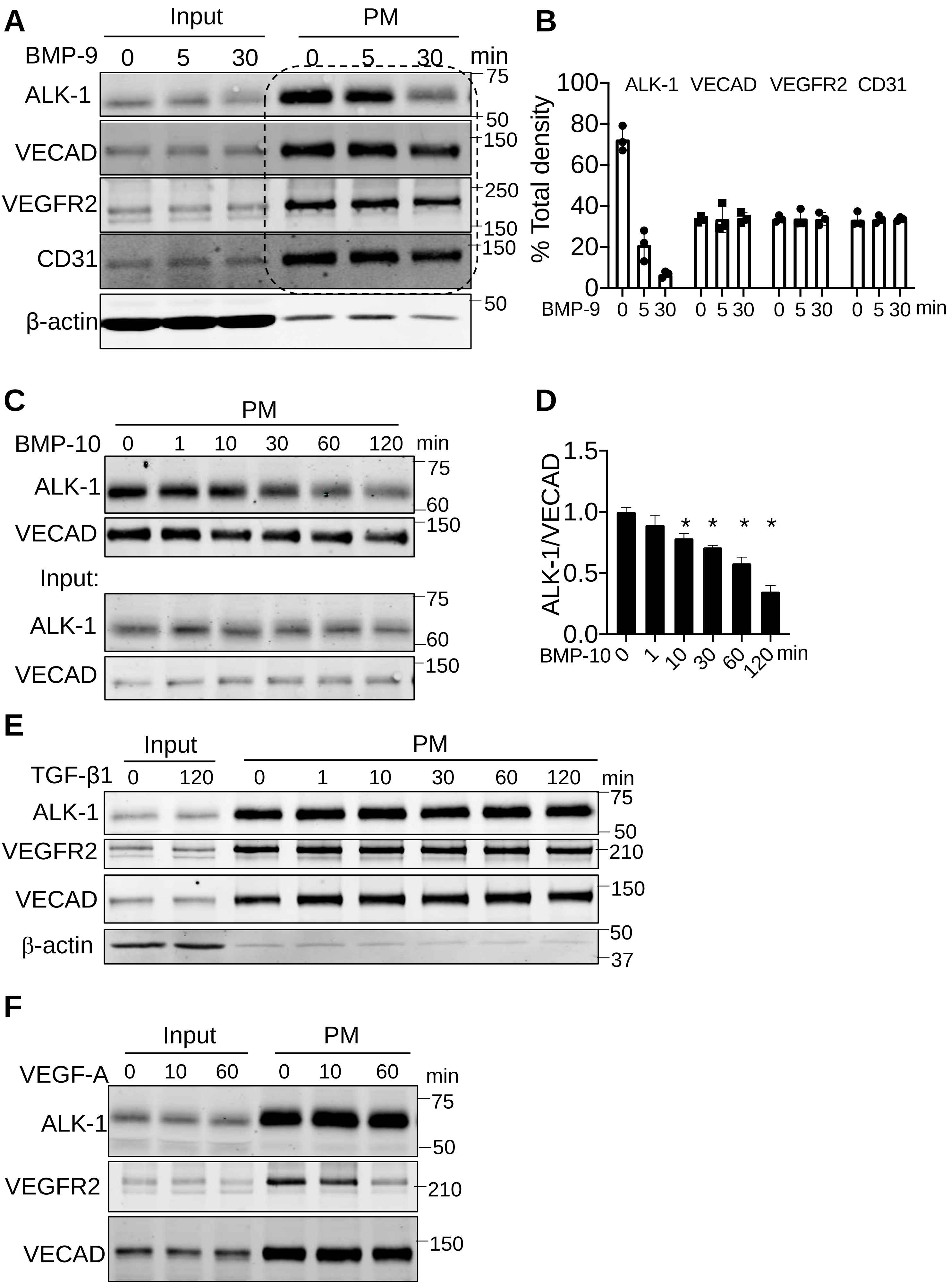

Supp Figure 2

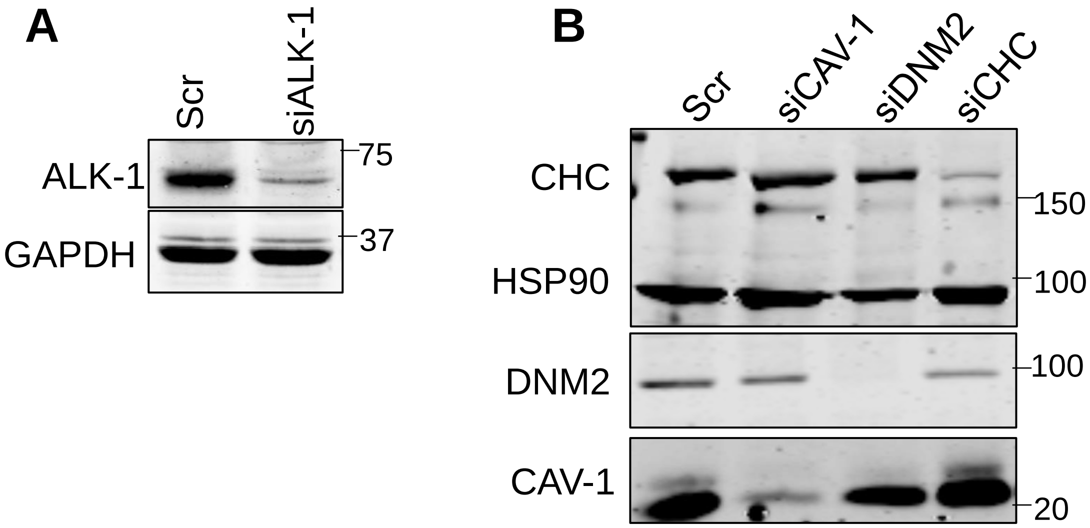

Supp Figure 3

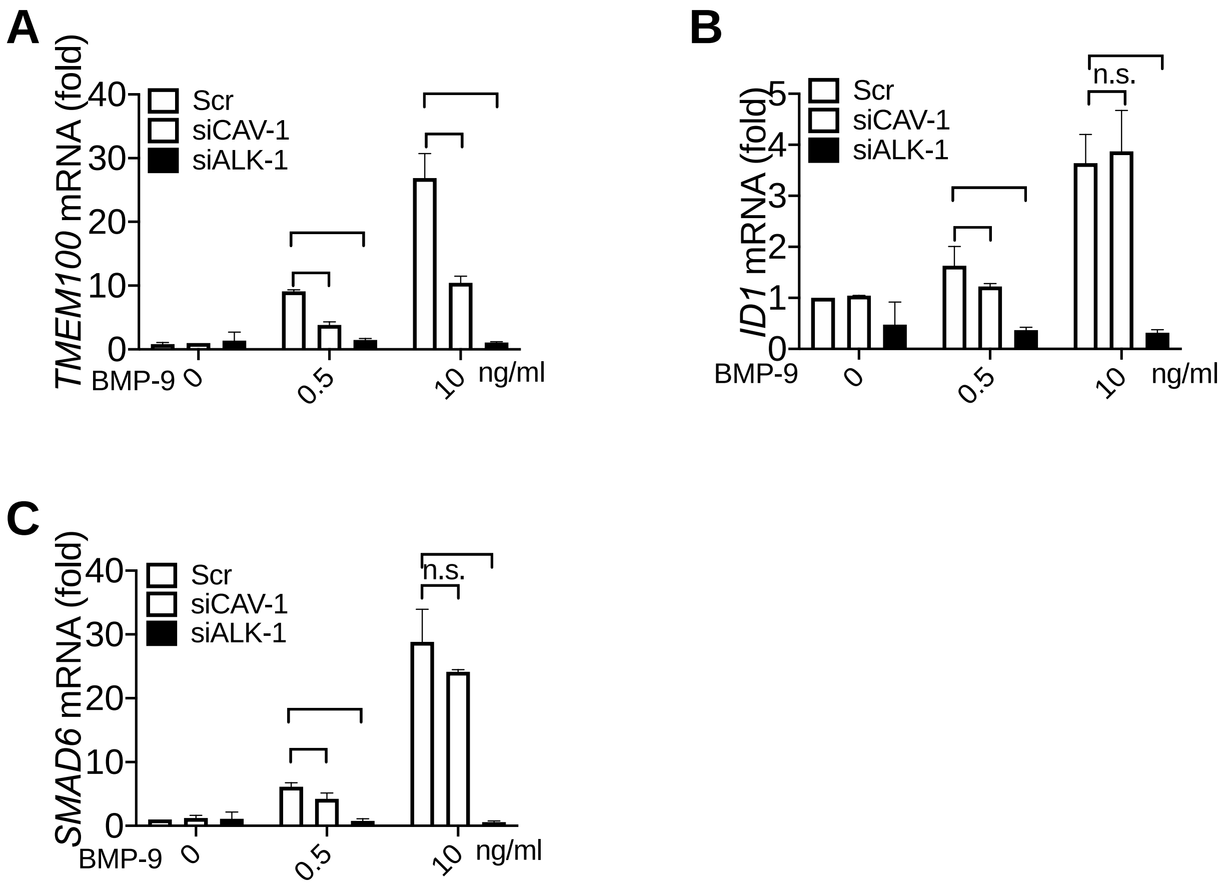

Supp Figure 4

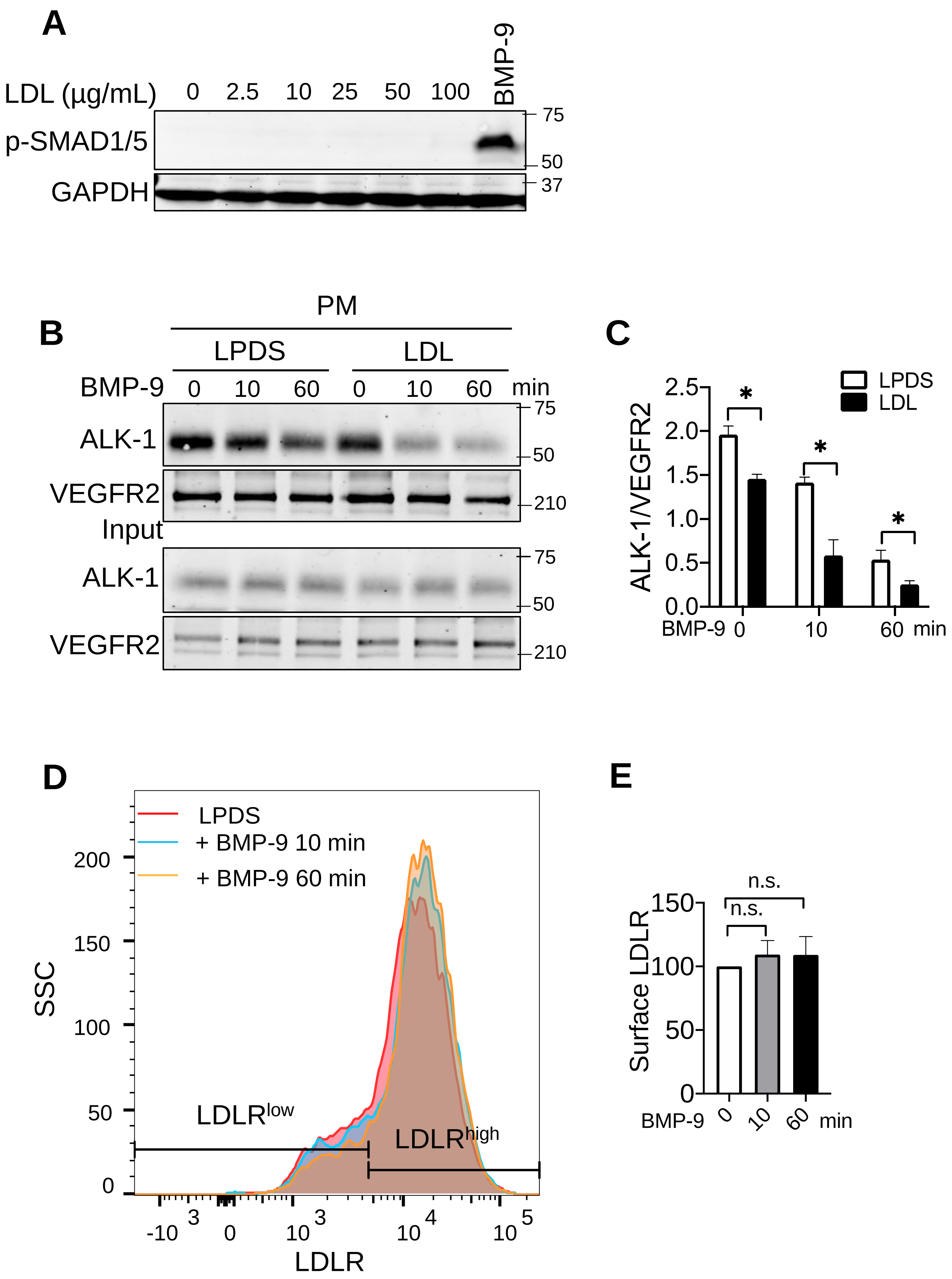

Supplement: Supplementary file 1 [file mmc1.pdf]
